# Supplementary material for: Modeling glioblastoma heterogeneity as a dynamic network of cell states
Source: Mol Syst Biol. 2021 Sep 16;17(9):e10105. doi: 10.15252/msb.202010105 (PMC8444284; doi:10.15252/msb.202010105)
Supplement: Supplementary file 6 — Source Data for Figure 5 [file MSB-17-e10105-s004.zip › Figure5A_sourcedata/GSEA_3017/hallmarks_stateF.GseaPreranked.1621934628066/HALLMARK_P53_PATHWAY.html]

Details for gene set HALLMARK\_P53\_PATHWAY[GSEA]

|  || Dataset | state33017 |
| Phenotype | NoPhenotypeAvailable |
| Upregulated in class | na\_pos |
| GeneSet | HALLMARK\_P53\_PATHWAY |
| Enrichment Score (ES) | 0.5426149 |
| Normalized Enrichment Score (NES) | 3.10588 |
| Nominal p-value | 0.0 |
| FDR q-value | 0.0 |
| FWER p-Value | 0.0 |
Table: GSEA Results Summary

  

Fig 1: Enrichment plot: HALLMARK\_P53\_PATHWAY      
 Profile of the Running ES Score & Positions of GeneSet Members on the Rank Ordered List

  

| PROBE | GENE SYMBOL | GENE\_TITLE | RANK IN GENE LIST | RANK METRIC SCORE | RUNNING ES | CORE ENRICHMENT || 1 | CDKN1A |  |  | 4 | 2.089 | 0.0546 | Yes |
| 2 | ZMAT3 |  |  | 10 | 1.568 | 0.0947 | Yes |
| 3 | MDM2 |  |  | 12 | 1.421 | 0.1326 | Yes |
| 4 | RPS27L |  |  | 33 | 1.025 | 0.1511 | Yes |
| 5 | MXD4 |  |  | 39 | 0.987 | 0.1755 | Yes |
| 6 | SAT1 |  |  | 45 | 0.957 | 0.1990 | Yes |
| 7 | BAX |  |  | 50 | 0.933 | 0.2224 | Yes |
| 8 | PLK2 |  |  | 77 | 0.805 | 0.2322 | Yes |
| 9 | APP |  |  | 95 | 0.759 | 0.2449 | Yes |
| 10 | FDXR |  |  | 120 | 0.716 | 0.2533 | Yes |
| 11 | BTG2 |  |  | 124 | 0.708 | 0.2710 | Yes |
| 12 | PHLDA3 |  |  | 131 | 0.696 | 0.2871 | Yes |
| 13 | HMOX1 |  |  | 142 | 0.684 | 0.3010 | Yes |
| 14 | DDB2 |  |  | 143 | 0.682 | 0.3194 | Yes |
| 15 | TOB1 |  |  | 161 | 0.646 | 0.3290 | Yes |
| 16 | PVT1 |  |  | 171 | 0.629 | 0.3419 | Yes |
| 17 | NINJ1 |  |  | 182 | 0.610 | 0.3538 | Yes |
| 18 | TRIAP1 |  |  | 208 | 0.584 | 0.3581 | Yes |
| 19 | HSPA4L |  |  | 215 | 0.579 | 0.3710 | Yes |
| 20 | DDIT4 |  |  | 216 | 0.579 | 0.3866 | Yes |
| 21 | BTG1 |  |  | 222 | 0.574 | 0.3998 | Yes |
| 22 | FBXW7 |  |  | 272 | 0.537 | 0.3919 | Yes |
| 23 | FAM162A |  |  | 282 | 0.524 | 0.4019 | Yes |
| 24 | CCNG1 |  |  | 292 | 0.515 | 0.4117 | Yes |
| 25 | NUPR1 |  |  | 295 | 0.513 | 0.4247 | Yes |
| 26 | CTSF |  |  | 301 | 0.510 | 0.4362 | Yes |
| 27 | TM7SF3 |  |  | 304 | 0.510 | 0.4490 | Yes |
| 28 | ISCU |  |  | 325 | 0.498 | 0.4533 | Yes |
| 29 | ABAT |  |  | 362 | 0.483 | 0.4499 | Yes |
| 30 | IP6K2 |  |  | 369 | 0.481 | 0.4601 | Yes |
| 31 | RAP2B |  |  | 372 | 0.480 | 0.4722 | Yes |
| 32 | CYFIP2 |  |  | 374 | 0.480 | 0.4847 | Yes |
| 33 | SESN1 |  |  | 384 | 0.476 | 0.4934 | Yes |
| 34 | PLXNB2 |  |  | 398 | 0.471 | 0.5002 | Yes |
| 35 | AK1 |  |  | 399 | 0.470 | 0.5129 | Yes |
| 36 | SLC3A2 |  |  | 411 | 0.466 | 0.5205 | Yes |
| 37 | XPC |  |  | 425 | 0.461 | 0.5270 | Yes |
| 38 | DDIT3 |  |  | 447 | 0.451 | 0.5295 | Yes |
| 39 | EPHX1 |  |  | 456 | 0.448 | 0.5380 | Yes |
| 40 | TSC22D1 |  |  | 473 | 0.443 | 0.5426 | Yes |
| 41 | DRAM1 |  |  | 519 | 0.427 | 0.5336 | No |
| 42 | ANKRA2 |  |  | 550 | 0.415 | 0.5310 | No |
| 43 | FOS |  |  | 592 | 0.401 | 0.5231 | No |
| 44 | IER5 |  |  | 605 | 0.396 | 0.5283 | No |
| 45 | PERP |  |  | 610 | 0.395 | 0.5371 | No |
| 46 | RGS16 |  |  | 691 | 0.373 | 0.5106 | No |
| 47 | FAS |  |  | 740 | 0.361 | 0.4984 | No |
| 48 | TSPYL2 |  |  | 748 | 0.359 | 0.5049 | No |
| 49 | FOXO3 |  |  | 801 | 0.346 | 0.4904 | No |
| 50 | PPM1D |  |  | 856 | 0.334 | 0.4747 | No |
| 51 | CD81 |  |  | 947 | 0.318 | 0.4421 | No |
| 52 | TRAF4 |  |  | 956 | 0.317 | 0.4470 | No |
| 53 | GADD45A |  |  | 1028 | 0.305 | 0.4228 | No |
| 54 | SLC7A11 |  |  | 1131 | 0.288 | 0.3838 | No |
| 55 | RNF19B |  |  | 1148 | 0.285 | 0.3842 | No |
| 56 | PIDD1 |  |  | 1218 | 0.277 | 0.3601 | No |
| 57 | PTPRE |  |  | 1243 | 0.275 | 0.3566 | No |
| 58 | CTSD |  |  | 1245 | 0.275 | 0.3635 | No |
| 59 | AEN |  |  | 1260 | 0.273 | 0.3645 | No |
| 60 | ABHD4 |  |  | 1313 | 0.267 | 0.3479 | No |
| 61 | TAP1 |  |  | 1317 | 0.266 | 0.3537 | No |
| 62 | KIF13B |  |  | 1325 | 0.265 | 0.3576 | No |
| 63 | SDC1 |  |  | 1438 | 0.254 | 0.3132 | No |
| 64 | HBEGF |  |  | 1583 | -0.282 | 0.2549 | No |
| 65 | ZFP36L1 |  |  | 1590 | -0.284 | 0.2599 | No |
| 66 | DCXR |  |  | 1689 | -0.315 | 0.2235 | No |
| 67 | UPP1 |  |  | 1726 | -0.328 | 0.2159 | No |
| 68 | TPRKB |  |  | 1944 | -0.450 | 0.1288 | No |
| 69 | TM4SF1 |  |  | 1948 | -0.452 | 0.1396 | No |
Table: GSEA details [plain text format]

  

Fig 2: HALLMARK\_P53\_PATHWAY: Random ES distribution      
 Gene set null distribution of ES for **HALLMARK\_P53\_PATHWAY**
